# Unusual presentation of cat scratch disease: case report

**DOI:** 10.1007/s10096-024-04872-1

**Published:** 2024-06-24

**Authors:** Sevgi Aslan Tuncay, Gulsen Akkoc, Seyhan Yilmaz, Aylin Dizi Isik, Pinar Canizci Erdemli, Burcu Parlak, Didem Buyuktas Aytac, Meryem Cagla Abaci Capar, Eda Almus, Ozge Yapici, Beyza Binici, Cigdem Ataizi Celikel, Sevliya Ocal Demir

**Affiliations:** 1 Department of Pediatrics, Division of Pediatric Infectious Diseases, Faculty of Medicine, Marmara University, Fevzi Cakmak, Muhsin Yazicioglu St, No:10, Floor: 6, Istanbul, Turkey; 2 Department of Radiology, Faculty of Medicine, Marmara University, Istanbul, Turkey; 3 Department of Medical Pathology, Faculty of Medicine, Marmara University, Istanbul, Turkey

**Keywords:** Lymphadenopathy, Osteomyelitis, Scratch, Disseminated

## Abstract

Cat scratch disease (CSD) is an infection caused by *Bartonella henselae*, presents with non-specific symptoms like lymphadenopathy, fever, and fatigue. It can progress to disseminated disease, leading to complications such as liver and splenic micro abscesses, osteomyelitis, encephalitis, and uveitis. Diagnosis is challenging due to varied presentations and limited tests. Treatment involves supportive care, with severe cases requiring antimicrobial therapy. In this report, we present a case of Cat scratch disease characterized by an atypical clinical manifestation, hepatosplenic and paravertebral involvement.

## Introduction

Cat scratch disease (CSD) is an infection caused by the gram-negative bacteria *Bartonella henselae*, belonging to the class Proteobacteria. Cats serve as natural reservoirs of the bacteria, and transmission to humans occurs through scratches, bites, or licks, particularly from infected kittens. Typically, CSD manifests with localized skin lesions and lymph node swelling. The incidence is higher during fall and winter, with a predilection for children aged five to nine years. Common symptoms include pustular skin lesions, swollen lymph nodes, and mild fever [1]. However, less common presentations encompass endocarditis, encephalopathy, osteolytic lesions, pneumonia, and ocular manifestations [2]. Treatment guidelines for disseminated pediatric cases remain undefined.

### Case

A previously healthy three-year-old boy presented to the emergency department with fever, abdominal pain, weight loss, and night sweats over the last 15 days. He was born in Ethiopia and moved to Turkey six months ago. There was no history of traveling to rural areas, tuberculosis contact, consumption of unpasteurized dairy products, fly bites, or tick bites. They had kittens at home. Childhood immunizations had been completed. Physical examination revealed left cervical and bilateral inguinal subcentimeter lymphadenopathies. There was diffuse abdominal tenderness.

**Fig. 1:**
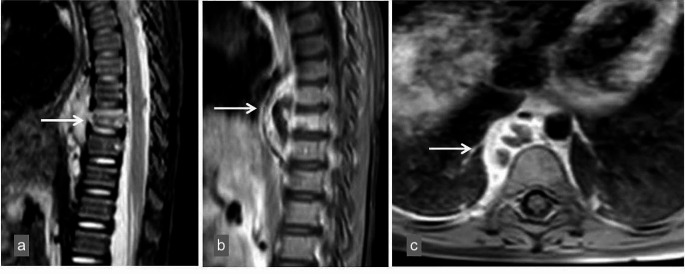
T2-weighted sagittal images (**a**) showed increased signal and loss of height in the 9th thoracic vertebra (arrow). Contrast-enhanced T1-weighted sagittal (**b**) and axial images (**c**) showed increased contrast enhancement in the 9th thoracic vertebra, a heterogeneous densely contrasted soft tissue lesion (abscess) with a cystic-necrotic component extending to the upper and lower adjacent prevertebral space (arrow)

Laboratory investigations revealed markedly elevated C-reactive protein (CRP) of 152 mg/L (0–5) and erythrocyte sedimentation rate of 84 mm/h (0–20). The blood count showed leukocytosis with a neutrophil predominance, with white blood cells (WBC) of 20.3 × 10³/µL (4.0–10.0), neutrophils of 13.5 × 10³/µL (1.4–6.2), and lymphocytes of 4.5 × 10³/µL (1.2–3.1). Liver function tests, renal function tests and serum electrolytes were normal. Abdominal ultrasonography revealed multiple, diffusely located millimetric anechoic lesions in the liver and spleen. Suspecting intra-abdominal bacterial infection, intravenous (IV) metronidazole and ceftriaxone treatments were initiated. A contrast-enhanced abdominal magnetic resonance imaging (MRI) was performed to further evaluate the lesions, revealing multiple subcentimeter lesions in the liver and spleen, as well as lymph nodes at the level of the portal hilus. Furthermore, a 3 × 1 cm lesion in the right paravertebral area at the Thoracal (T) 10–12 level was identified with heterogeneous contrast enhancement in the T10 vertebral corpus. Vertebral MRI demonstrated a contrast-enhanced lesion characterized by septa-like structures in the T9-11 paravertebral region, measuring 13 mm in thickness and 50 mm in length. Additionally, there was contrast and height loss in the T10 vertebral body (Fig. 1). It was suspected that the involvement was due to an underlying infection, and therefore diagnostic tests were performed. As the fever persisted, the patient’s treatment was changed to IV teicoplanin and meropenem.

The thorax computed tomography (CT) examination and echocardiography were normal. The tuberculin skin test was nonreactive. Additionally, acid-fast bacilli and *M. tuberculosis complex* polymerase chain reaction (PCR) were not detected in the consecutive three fasting gastric fluids. The thick smear analyzed during fever did not show any Leishmania and Plasmodium trophozoites. Serologic tests for Epstein-Barr Virus, Cytomegalovirus, Human Immunodeficiency Virus, *Brucella spp*, *Francisella tularensis*, *Coxiella burnetti*, *Bartonella henselae*, and *Echinococcus granulosus* were performed; all serological tests were negative except for Bartonella IgM and IgG, which were detected positive at titers of 1/100 and > 1/320, respectively. *Coxiella burnetti* IgM was positive, while IgG test was negative. A punch biopsy was performed on the liver. The patient was started on rifampicin, gentamicin, and azithromycin with the initial diagnosis of disseminated CSD. To ensure coverage of Q fever, azithromycin was discontinued on the fifth day and trimethoprim/ sulfamethoxazole (TMP/SMX) was added. For disseminated CSD and prolonged fever, 1 mg/kg methylprednisolone treatment was initiated for seven days, then tapered and discontinued within 21 days.

**Fig. 2:**
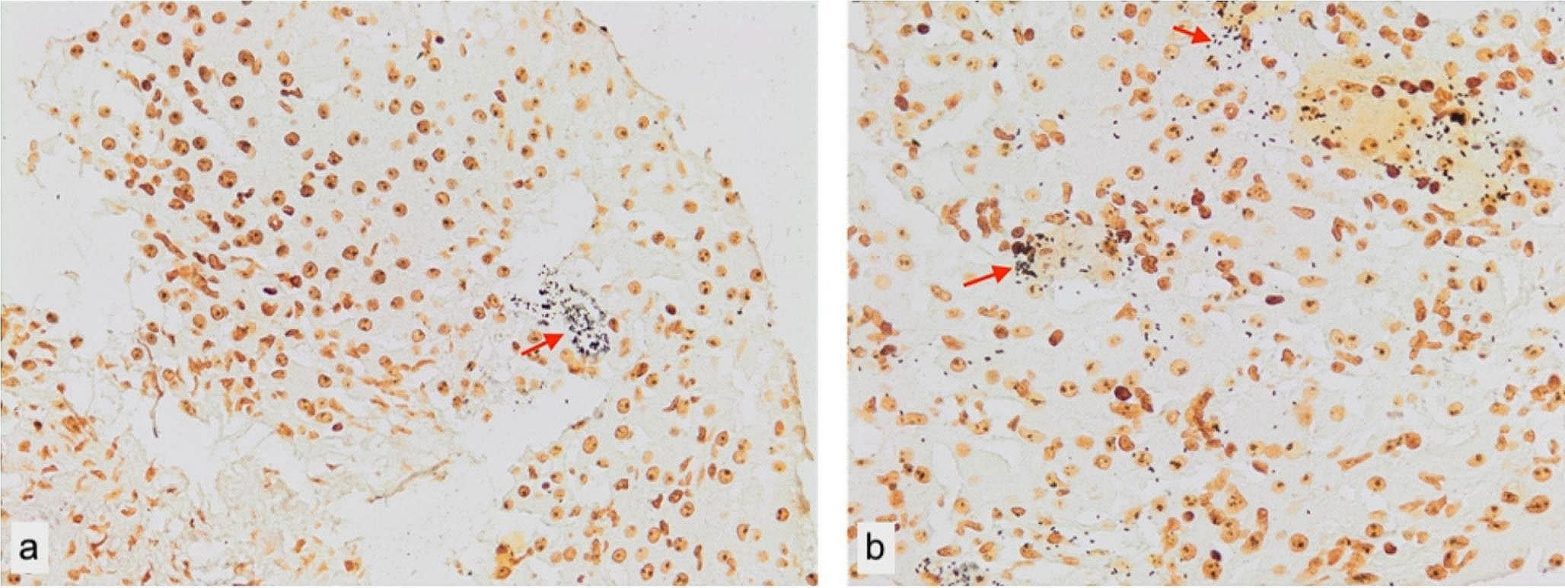
This image shows a light micrograph of Warthin-Starry silver–stained *B. henselae* bacteria. With Warthin-Starry stain, they appear as small, black-curved organisms either in clusters (**a**, arrow) or singly (**b**, arrow)

On the 16th day of hospitalization, the patient’s fever resolved. Confirmation tests for Q fever resulted negative. Gentamicin was completed in 21 days. The liver biopsy revealed intense inflammatory deposits, primarily consisting of lymphocytes, plasma cells, and a few neutrophil leukocytes. Inflammatory cells were distributed along the sinusoids, and secondary sinusoidal dilatation due to hepatocellular atrophy was observed in the parenchyma. Additionally, some areas showed sinusoidal dilatation foci with a peliosis hepatis-like pattern. Microorganisms were observed in Warthin-Starry staining (Fig. 2).

**Fig. 3:**
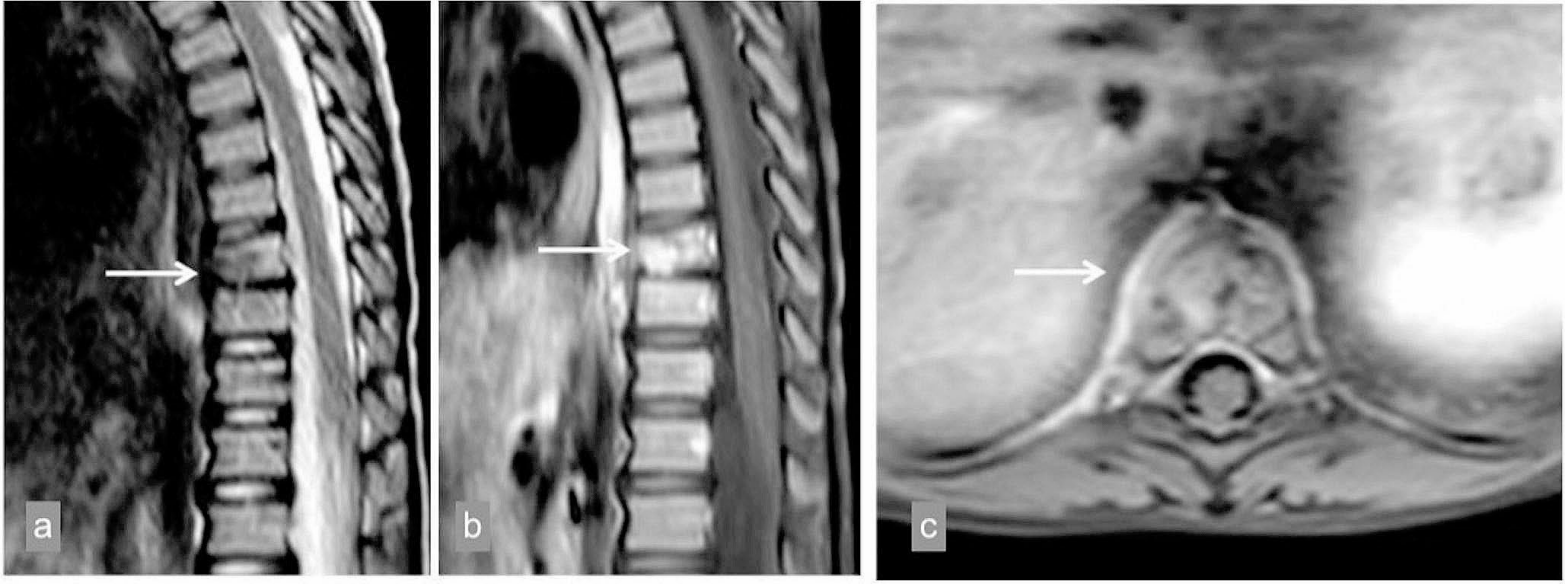
In the contrast-enhanced thoracic vertebra MRI examination taken after the treatment; Loss of height in the 9th thoracic vertebra in T2-weighted sagittal (**a**) images and increased contrast enhancement in the vertebra in contrast-enhanced T1-weighted sagittal (**b**) sections continue. However, in contrast-enhanced axial sections (**c**), regression was observed in the abscess formation extending to the prevertebral space (arrow)

Follow-up of the patient continued an outpatient basis with oral TMP/SMX and rifampicin. Pyogenic, fungal, and mycobacterial cultures of liver tissue yielded no growth. Contrast-enhanced vertebral MRI performed in the second month of treatment showed almost complete regression of the septate cystic lesion in the paravertebral area, but osteomyelitis findings in the vertebral bone persisted (Fig. 3). The patient’s treatment was planned to continue for six to twelve months.

## Discussion

In this report, we present a pediatric case wherein various viral, parasitic, and bacterial agents were examined to determine the etiology of prolonged fever alongside hepatosplenic and paravertebral lesions. Despite the absence of a history of cat scratching, the presence of kittens in the household was noted. Transmission of CSD usually occurs through scratches or bites from infected cats or through contact with fleas that have acquired the bacteria from cats. However, since there was no clear information about the patient’s contact with a flea, we could not comment on this issue.

Diagnosing CSD can pose a challenge, especially in cases with atypical organ involvement. Only 10% of patients with *B. henselae* infection develop hepatic granuloma or splenic abscess [3]. Osteomyelitis is even rarer, affecting only 0.27% of patients. Although it represents an uncommon manifestation, awareness of osteomyelitis associated with CSD is crucial. In cases involving bone, the organism spreads via hematogenous or lymphatic routes [4]. The differential diagnosis includes conditions with a less favorable prognosis, such as histiocytosis X, other granulomatous infections (e.g., tuberculosis, brucellosis), and metastatic malignancy [5]. 

Diagnostic tests for CSD include enzyme immunoassay (EIA) and indirect immunofluorescence assays (IFA) to detect IgM and IgG serum antibodies to *Bartonella species*. However, both methods have limitations in sensitivity and specificity [1]. The diagnostic tool with the highest sensitivity remains Bartonella PCR performed on lymph node biopsy or abscess aspirate [6]. However, Bartonella PCR assay is not generally recommended for testing blood specimens. Isolating *B. henselae* through routine culture requires prolonged incubation (one to four weeks), and isolating the bacteria is challenging. Therefore, *B. henselae*-specific serology remains the primary diagnostic tool [1]. If tissue specimens (e.g., lymph nodes) are available, histopathology typically reveals granulomatous infection with a necrotic center, and Warthin-Starry silver stain aids in identifying *B. henselae* as pleomorphic rod-shaped bacilli [2]. 

Cat Scratch Disease is a self-limited condition that typically resolves spontaneously within two to four months [6]. Antibiotics are generally unnecessary, and surgical excision should be avoided. Painful suppurative nodes can be managed with needle aspiration. Antimicrobial therapy is recommended for severely ill patients exhibiting systemic symptoms, as well as for all immunocompromised patients [1]. 

The optimal treatment for CSD remains uncertain. Arisoy et al. reported improvement in prolonged fever among patients with hepatosplenic CSD through combination therapy with TMP-SMX when rifampin was added to the regimen, typically administered for a duration of 14 days [7]. Doxycycline plus rifampin may be used for patients with neuroretinitis [1]. Treatment strategies for complicated cases, including osteomyelitis, may involve azithromycin, rifampin, ciprofloxacin, trimethoprim/sulfamethoxazole, or gentamicin as monotherapy or in combination [8]. The clinical relevance of in vitro susceptibility data to management remains uncertain. However, there are currently no formal guidelines regarding the treatment of disseminated pediatric cases with hepatosplenic lesions or osteomyelitis [9, 10]. Our patient was referred to the pediatric surgery department for potential excision of the paravertebral mass. Given the high risk and complexity associated with surgery in this area, initial recommendations leaned towards attempting diagnosis via liver biopsy. If the liver biopsy failed to yield adequate diagnostic insights or if there was no response to treatment, excision of the mass was considered as the subsequent step. Following the performance of a liver biopsy, the diagnosis of CSD was confirmed through serum serology, and treatment was promptly initiated. Surgery was not pursued as the patient exhibited a positive response to antibiotic therapy.

## Conclusion

This case underscores the importance of considering cat scratch disease as a differential diagnosis in cases of cat contact, even in the absence of a history of scratching, particularly in patients presenting with fever and a paravertebral mass of unknown etiology. Additionally, *B. henselae*-specific serology may offer crucial insights for accurately establishing the diagnosis of CSD.

## Data Availability

No datasets were generated or analysed during the current study.
